# Metagenomic analysis of intestinal microbiota characteristic differences between patients with ankylosing spondylitis and healthy individuals

**DOI:** 10.1186/s12866-026-04996-8

**Published:** 2026-04-06

**Authors:** Shuo-wen Liu, Xin-xin Wang, Le-yao Xian, Da-wei Zou, Yu-feng Huang, Xi-lin He, Fan He, Xiao-tong Wang

**Affiliations:** 1 The First Clinical College of Liaoning University of Traditional Chinese Medicine, Affiliated Hospital of Liaoning University of Traditional Chinese Medicine, Shenyang, Liaoning China; 2 Guangdong Provincial Hospital of Chinese Medicine, The Second Affiliated Hospital of Guangzhou University of Chinese Medicine, Guangzhou, Guangdong China; 3 University of Toronto, St. George Campus, Toronto, ON Canada

**Keywords:** Ankylosing spondylitis, Macrogenomics, Gut microbiota, Microbial community function, Clinical trial

## Abstract

**Background:**

To explore the differences in intestinal microbiota between patients with ankylosing spondylitis (AS) and healthy individuals (HC) in terms of genetic, species composition, and functional levels, and to reveal the role of intestinal microorganisms in the pathogenesis of AS.

**Methods:**

This study selected 17 AS patients (AS group) and 17 healthy subjects (HC group) from the Affiliated Hospital of Liaoning University of Traditional Chinese Medicine between August to October 2024. Basic clinical data, as well as the Bath Ankylosing Spondylitis Disease Activity Index (BASDAI), Visual Analogue Scale (VAS) score, of the AS group, were collected. Fresh fecal samples were also collected for metagenomic sequencing. Differences in microbiota were analyzed using methods including Alpha diversity analysis, species abundance analysis, Principal Coordinates Analysis (PCoA), Non-metric Multidimensional Scaling (NMDS), DESeq2 analysis, Linear Discriminant Analysis Effect Size (LEfSe), and Kyoto Encyclopedia of Genes and Genomes (KEGG) functional annotation.

**Results:**

The number of unique genes in the AS group (566,526) was higher than that in the HC group (406,609). At the species level, there were no significant differences in Alpha diversity or the overall microbial structure (revealed by PCoA and NMDS) between the two groups (*p* > 0.05). However, significant differences in abundance were observed at the family, genus, and species levels. DESeq2 identified a total of 43 differential species, among which 22 species had increased abundance and 21 species had decreased abundance in the AS group. LEfSe analysis showed that the HC group had 16 dominant bacterial species, while the AS group had only *Neoporus faecalis* as the dominant species. There were differences in KEGG Level 3 functional pathways between the two groups, but no statistically significant difference was found in the overall functional structure (*p* = 0.698). Functional enrichment analysis revealed that AS-specific genes were primarily enriched in neurodegenerative disease pathways, protein processing in the endoplasmic reticulum, and autophagy-related pathways, with substantial contributions from genera including *Bacteroides*, *Streptococcus*, *Eubacterium*, and *Faecalibacterium*. However, neither individual differential species nor their functional pathways showed significant correlations with clinical disease activity scores (BASDAI and VAS)。.

**Conclusion:**

The studies indicated that although there was no significant difference in the overall diversity of intestinal microbiota between AS patients and healthy individuals, there were obvious distinctions in genetic composition, specific bacterial species, and functional pathways, suggesting that intestinal microorganisms may be involved in the pathogenesis of AS.

**Supplementary Information:**

The online version contains supplementary material available at 10.1186/s12866-026-04996-8.

## Introduction

Ankylosing spondylitis (AS) is a chronic progressive autoimmune disease characterized by severe symptoms, a long course, and a lack of specific drugs. The incidence rate in China ranges from 0.2% to 0.4%, with a higher prevalence in males than in females [1]. Conventional medicine mainly uses non-steroidal anti-inflammatory drugs and anti-rheumatic drugs for treatment, which have limitations such as poor efficacy and many adverse reactions [2, 3].

The intestinal microbiome symbiotes with the host and affects the host’s metabolism, physiology, and immunity. A variety of chronic autoimmune diseases are associated with intestinal microbiota imbalance, and AS is no exception [4–6]. Studies have confirmed that the intestines of AS patients contain adherent and invasive bacteria, with a high detection rate of *Klebsiella pneumoniae*, which is associated with disease activity. More than 70% of AS patients have subclinical intestinal inflammation, and some may develop inflammatory bowel disease [7], the increase in serum total IgA in patients with active disease suggests intestinal barrier problems [8]. Animal experiments have shown that HLA-B27 transgenic mice do not develop the disease in a germ-free environment, but develop the disease after exposure to intestinal bacteria; changes in fecal microbiota are associated with HLA-B27, indicating that intestinal microbiota plays a key role in the pathogenesis of AS [9, 10] and may be a potential target for treatment. Therefore, the characterization of the species composition of the microbiome and the exact mechanism of the functional role of the intestinal microbiome in disease progression are the focus of recent research. The study on the regulation of the immune system of AS patients by intestinal microbiota needs further exploration to provide more powerful evidence and breakthrough points for disease prevention and treatment.

Although existing studies suggest an association between intestinal microbiota and AS, most focus on analyzing the microbiota composition using 16 S rRNA gene sequencing technology [11], which makes it difficult to fully analyze genetic differences and functional characteristics. Metagenomic sequencing technology can directly sequence the genomes of all microorganisms in the environment, which not only accurately identifies the species composition but also in-depth analyzes gene functions and metabolic pathways, providing a more comprehensive perspective for revealing the association mechanism between intestinal microbiota and diseases [12].

This study used metagenomic sequencing technology to explore the structure and functional characteristics of intestinal microbiota between AS patients and healthy individuals, in order to provide a basis for revealing the pathogenesis of AS and guiding clinical diagnosis and treatment.

## Materials and methods

### Subject recruitment

A total of 17 AS patients who visited the Rheumatology Clinic of the Affiliated Hospital of Liaoning University of Traditional Chinese Medicine from August to October 2024 were collected; meanwhile, 17 healthy subjects were recruited from the Physical Examination Center of the Affiliated Hospital of Liaoning University of Traditional Chinese Medicine during the same period. These criteria aim to avoid the impact of other diseases, medications, or dietary habits on the microbiota and metabolites. Detailed inclusion and exclusion criteria for patients and healthy controls are provided in the supplementary materials. For the collection of fresh fecal samples from the subjects, they were required to empty their bladders before sampling. A sterile fecal spoon was used to collect 3 g of samples from 3 internal parts of the feces. After sampling, the samples were frozen in liquid nitrogen within 30 min and transferred to a -80 °C refrigerator for storage at 4 h later, which were used for fecal metagenomic sequencing. Metagenomic DNA extraction and sequencing analysis of fecal samples were completed in October 2024.This study was approved by the Medical Ethics Committee of the Affiliated Hospital of Liaoning University of Traditional Chinese Medicine (approval number: AF/SW-09/06.2).

### DNA extraction and metagenomic sequencing

Fecal sample DNA from 17 AS patients and 17 healthy controls was subjected to sequencing of 300–400 bp fragment libraries using the NovaSeq sequencing platform (Illumina, USA). After quality control of the raw data with fastp to obtain clean reads, Bowtie2 was used to remove host sequences, resulting in high-quality reads with host sequences eliminated.

Subsequently, MEGAHIT was applied for the de novo assembly of these reads. For scaffolds with a length of ≥ 500 bp, MetaGeneMark was used for open reading frame (ORF) prediction, and CD-HIT software was employed for deduplication. Finally, a non-redundant gene set (unigenes) was obtained.

Based on this non-redundant gene set, the host-removed reads were aligned to the unigenes using Bowtie2. Low-abundance genes with aligned read count ≤ 2 were filtered out, thereby generating a gene abundance table for subsequent analysis. Meanwhile, DIAMOND was used to align the unigenes with the KEGG database for functional annotation, and the LCA (Lowest Common Ancestor) algorithm was adopted to align the unigenes with the Micro_NR database for species annotation.

### Microbiome statistical analysis

To obtain the relative abundance of bacteria, the abundance of different bacterial species was divided by the total number of bacteria in each sample.

Principal Coordinates Analysis (PCoA) based on Bray-Curtis distance and Permutational Multivariate Analysis of Variance (PERMANOVA) were used to compare the microbial profiles between the healthy control (HC) group and the ankylosing spondylitis (AS) group. The Bray-Curtis distance is a distance metric for calculating community similarity, which takes characteristic abundance data into account in the calculation. The sample distance reflects the similarity of species/functional composition structure: the closer the distance, the higher the similarity; the farther the distance, the greater the difference.

DESeq2 analysis is a differential expression analysis method based on the negative binomial distribution, widely applied for statistical modeling and hypothesis testing of high-throughput sequencing data. This approach enables more rapid identification of species or functional features with significantly different abundances between groups.

Linear Discriminant Analysis Effect Size (LEfSe) analysis was used to screen for biomarkers between the HC group and the AS group. LEfSe analysis combines non-parametric tests and Linear Discriminant Analysis (LDA), with the LDA screening threshold set to 2. An LDA value > 2 indicates that the difference is statistically significant.

The correlation analysis between AS clinical phenotypes and differential microbiota was conducted on the BioinCloud platform (https://www.bioincloud.tech) [13].

### Experiment statistical analysis

SPSS 27.0 was used for statistical analysis. Measurement data conforming to normal distribution were described as mean ± standard deviation ($\:\mathrm{x}̄$±s), otherwise as median (quartile) [M (P25, P75)]. Independent samples t-test (normal distribution and homogeneous variance), corrected t-test (normal distribution but heterogeneous variance), or Wilcoxon rank-sum test (non-normal distribution) were used for comparison between the two groups. A *p*-value < 0.05 was considered statistically significant.

## Results

### Comparison of general information

A total of 17 cases in the HC group and 17 cases in the AS group were included in the study. As shown in Table 1, there were no statistically significant differences in age, gender, height, or weight between the two groups of subjects (*p* > 0.05).

**Table 1:** Patient and healthy individual basic information form

Project	AS (*n* = 17)	HC (*n* = 17)	*p*-Value
Age/year, *M* (P_25_, P_75_)	37 (35.5, 56.5)	45 (29.0, 41.5)	0.099
Gender (male/female)/case	9/8	7/10	0.563
Height /cm, *M* (P_25_, P_75_)	172 (161.35, 179.60)	163 (160, 173)	0.394
Weight /kg, *M* (P_25_, P_75_)	65 (59.0, 73.5)	61 (57.5, 69.5)	0.518
BASDAI ($\overline{x}\pm SD$)	4.21 ± 0.47	NA	NA
VAS	57.06 ± 6.05	NA	NA

### Analysis of the number of intestinal microbiota genes

**Fig. 1:**
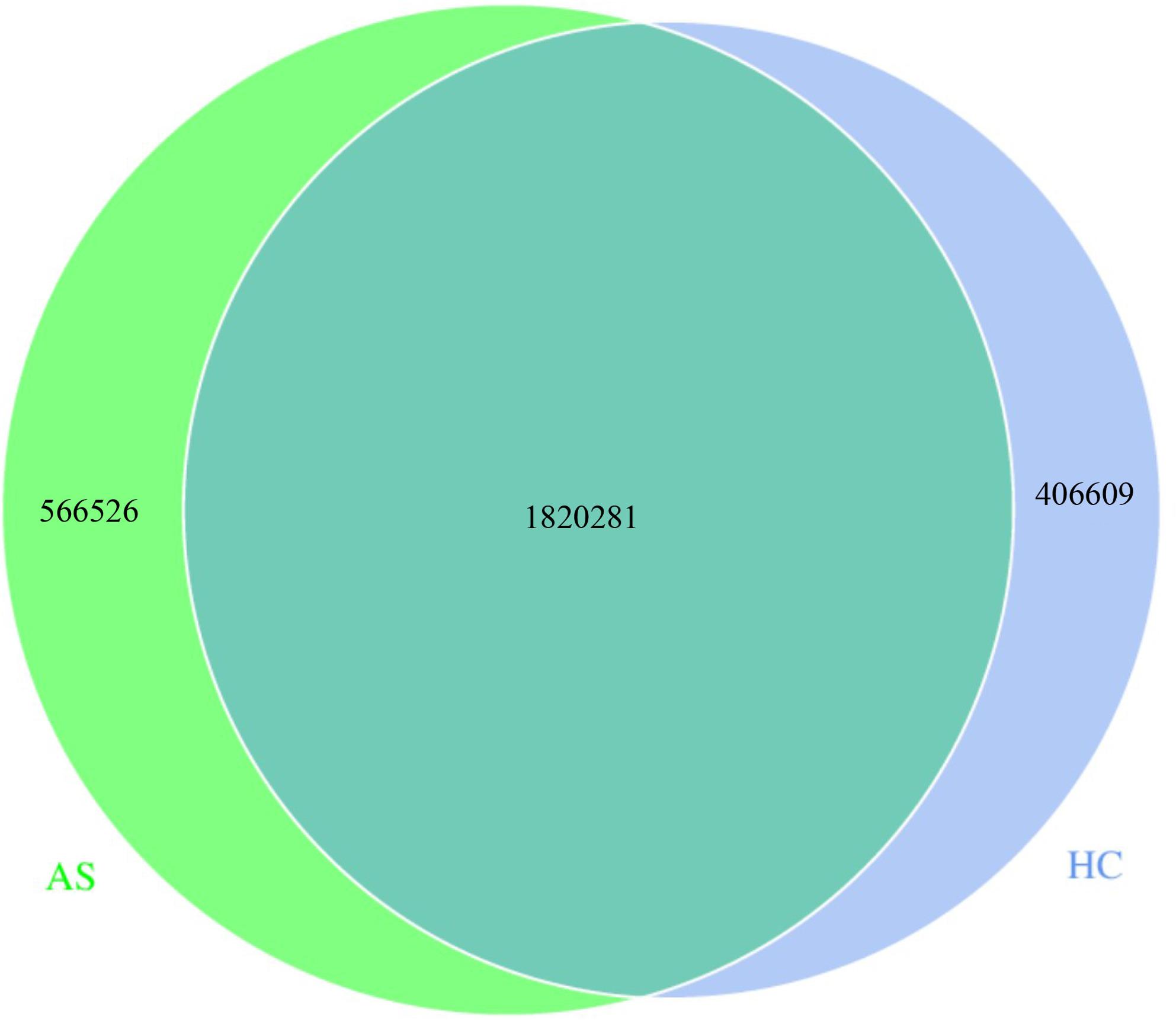
Comparison of the number of genes between groups. HC: Healthy Control group; AS: Ankylosing Spondylitis group

The number of unique genes in the AS group was 566,526, that in the HC group was 406,609, and the number of common genes between the two groups was 1,820,281 (Fig. 1).

### Analysis of intestinal microbiota composition

#### Analysis of species alpha diversity index

**Fig. 2:**
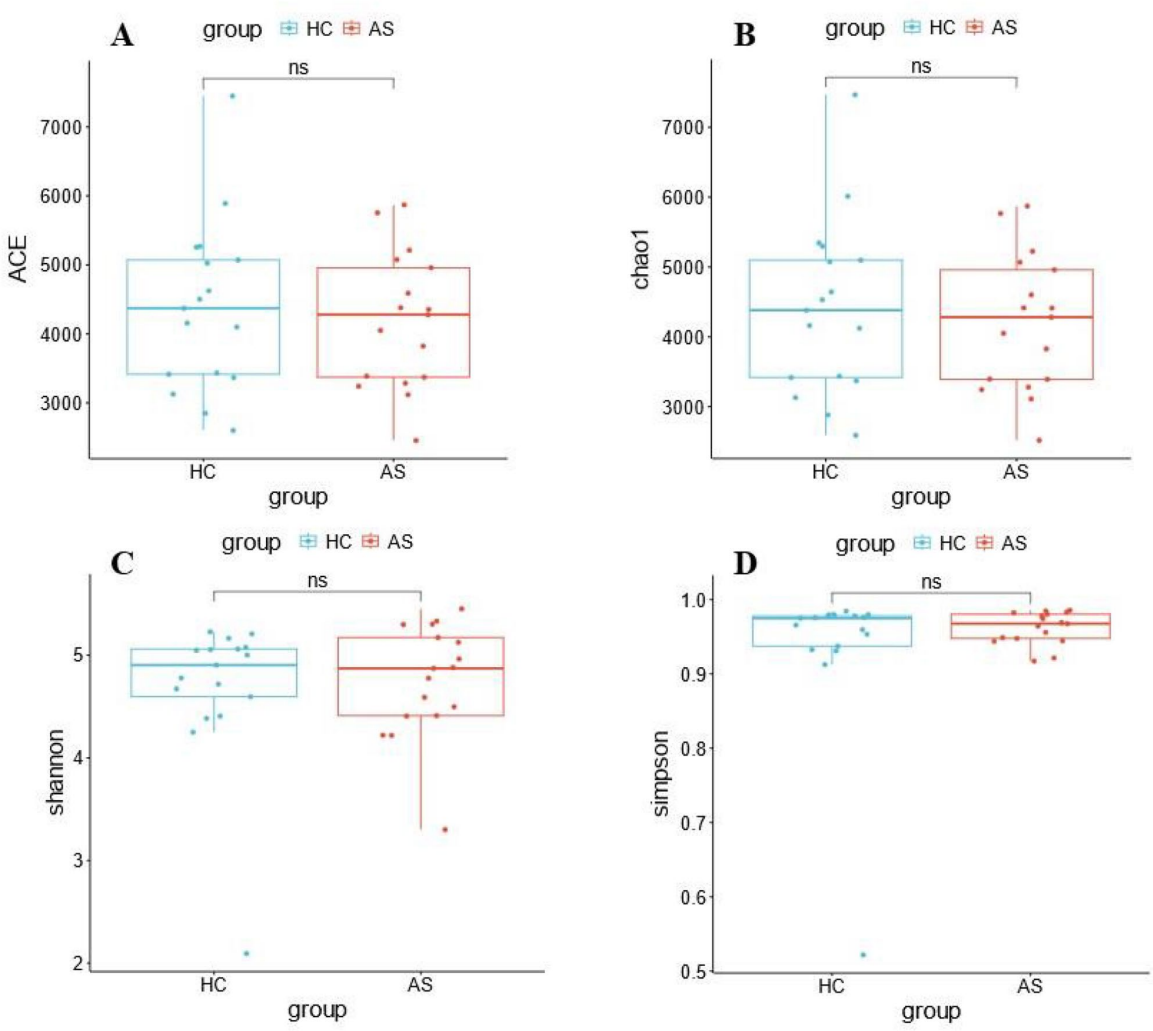
Analysis of fecal metagenomic species-level Alpha diversity indices between AS patients and healthy individuals. **A** ACE analysis; **B** Chao1 analysis; **C** Shannon analysis; **D** Simpson analysis

Alpha diversity analysis was conducted on each classification level. At the species level, the Chao1 index (reflecting community richness), Shannon index, and Simpson index (all reflecting community diversity) were used to analyze the results, which showed that there was no statistically significant difference in the richness and diversity of microbial communities between groups (*p* > 0.05) (Fig. 2).

#### Overview of species relative abundance

**Fig. 3:**
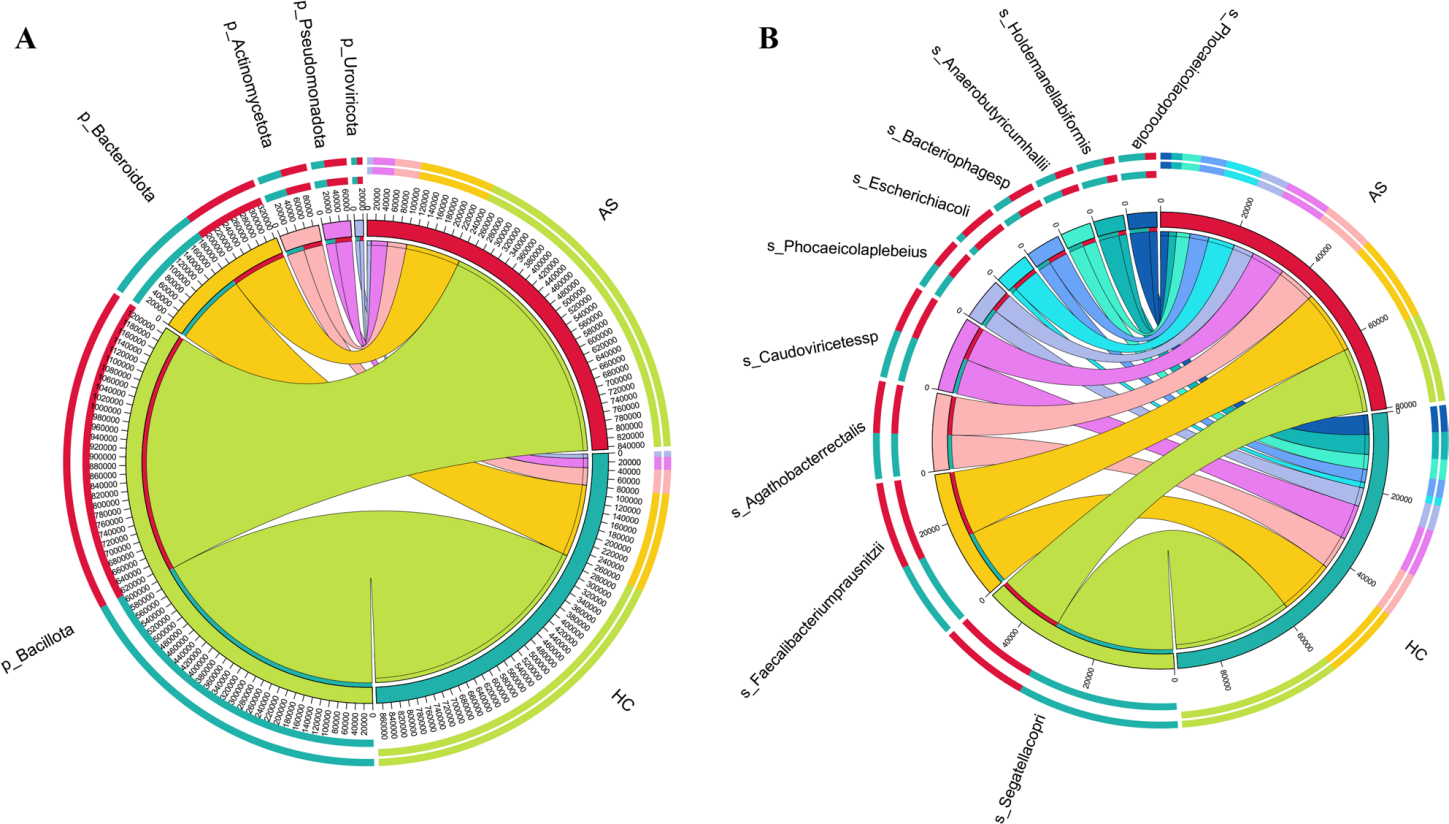
Relative abundance of fecal metagenomic species between AS patients and healthy individuals. **A** Relative abundance of the top 5 species at the phylum level; **B** Relative abundance of the top 10 species at the species level. HC: healthy control group; AS: ankylosing spondylitis group

The species annotation results of intestinal microbiota showed that there were differences in the distribution of relative abundance of intestinal microbiota between the AS group and the HC group. The differences were small at the phylum, class, and order levels, while significant differences were observed at the family, genus, and species levels (Fig. 3).

At the phylum level, the main dominant phyla in both the HC and AS groups included *Bacillota*, *Bacteroidota*, *Actinomycota*, and *Pseudomonadota*. The abundance of *Pseudomonadota* in the AS group showed a trend of difference compared with that in the HC group, but the difference was not significant. At the species level, the dominant species included *Segatella copri*, *Faecalibacterium prausnitzii*, and *Agathobacter rectalis*. The species with the highest relative abundance in both the HC and AS groups was *Segatella copri*.

#### Species PCoA analysis and NMDS analysis

**Fig. 4:**
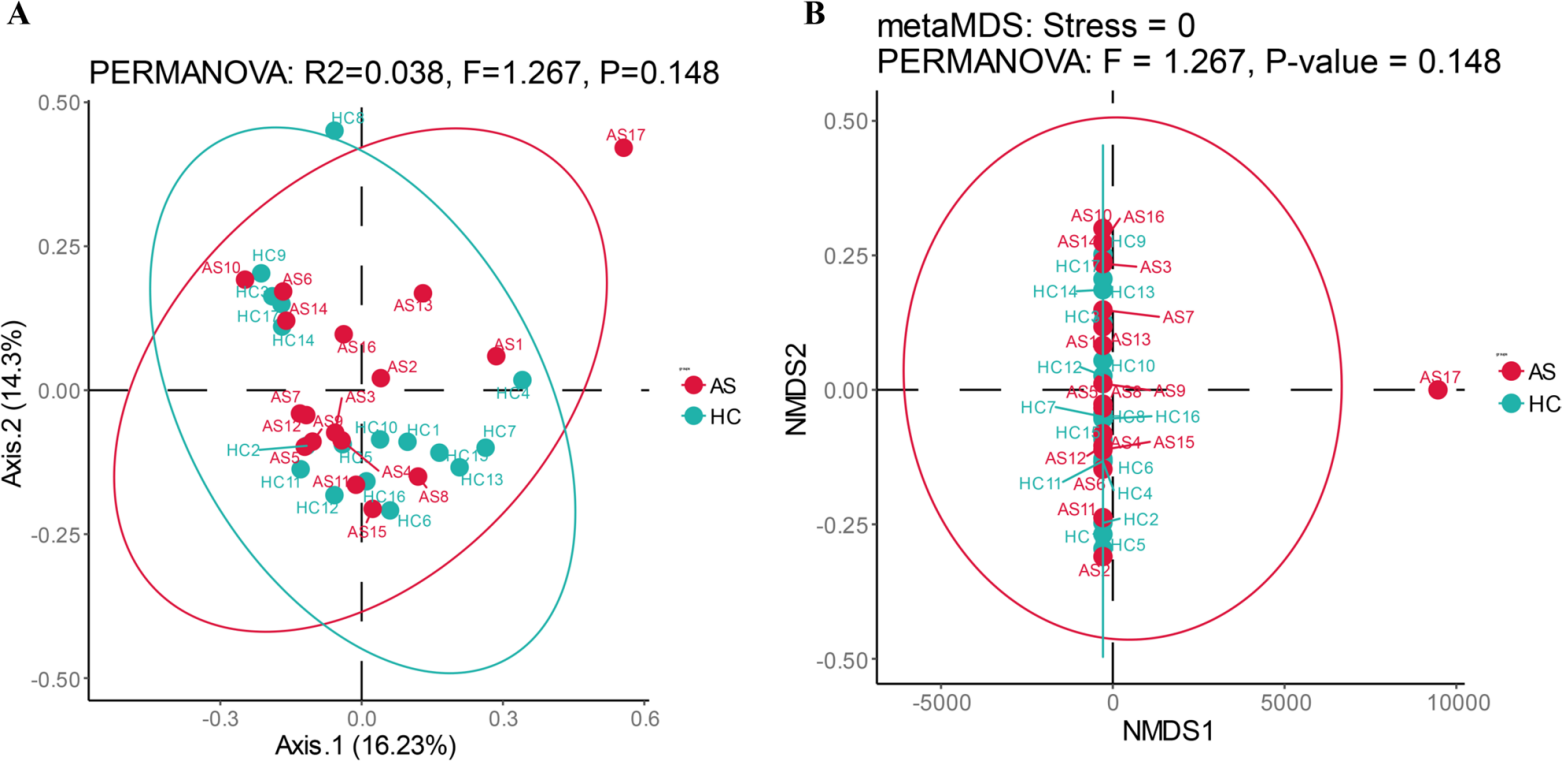
Analysis results of intestinal microbiota at the species level. **A** PCoA analysis; **B** NMDS analysis

PCoA analysis: PCoA analysis was conducted based on each classification level. The PCoA analysis method based on Bray-Curtis distance and PERMANOVA was used to analyze the microbial composition pattern. At the species level, the overlapping rate of the fitted circles between the AS group and the HC group was relatively high, with R²=0.038 and *P* = 0.148, indicating no significant difference between the samples (*p* > 0.05) (Fig. 4A).

#### Species NMDS analysis

NMDS analysis was conducted based on each classification level. Stress < 0.2 indicated that NMDS could accurately reflect the degree of difference between samples. At the species level, the overlapping rate of the fitted circles between the HC group and the AS group was relatively high, with *p*-value = 0.148, indicating no significant difference between the samples (Fig. 4B).

#### Species-level DESeq2 analysis

**Fig. 5:**
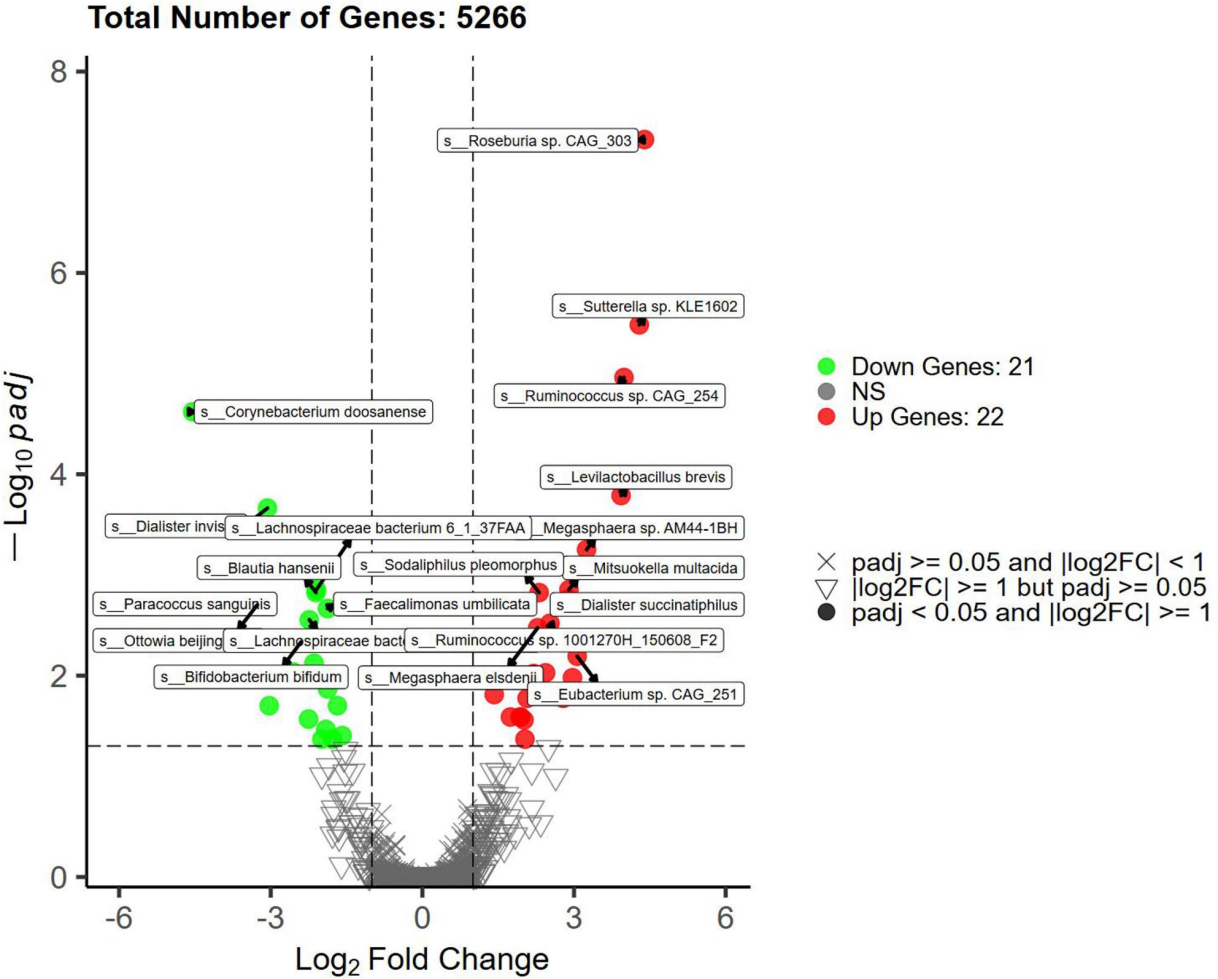
Species-Level DESeq2 Analysis Results of Gut Microbiota

**Fig. 6:**
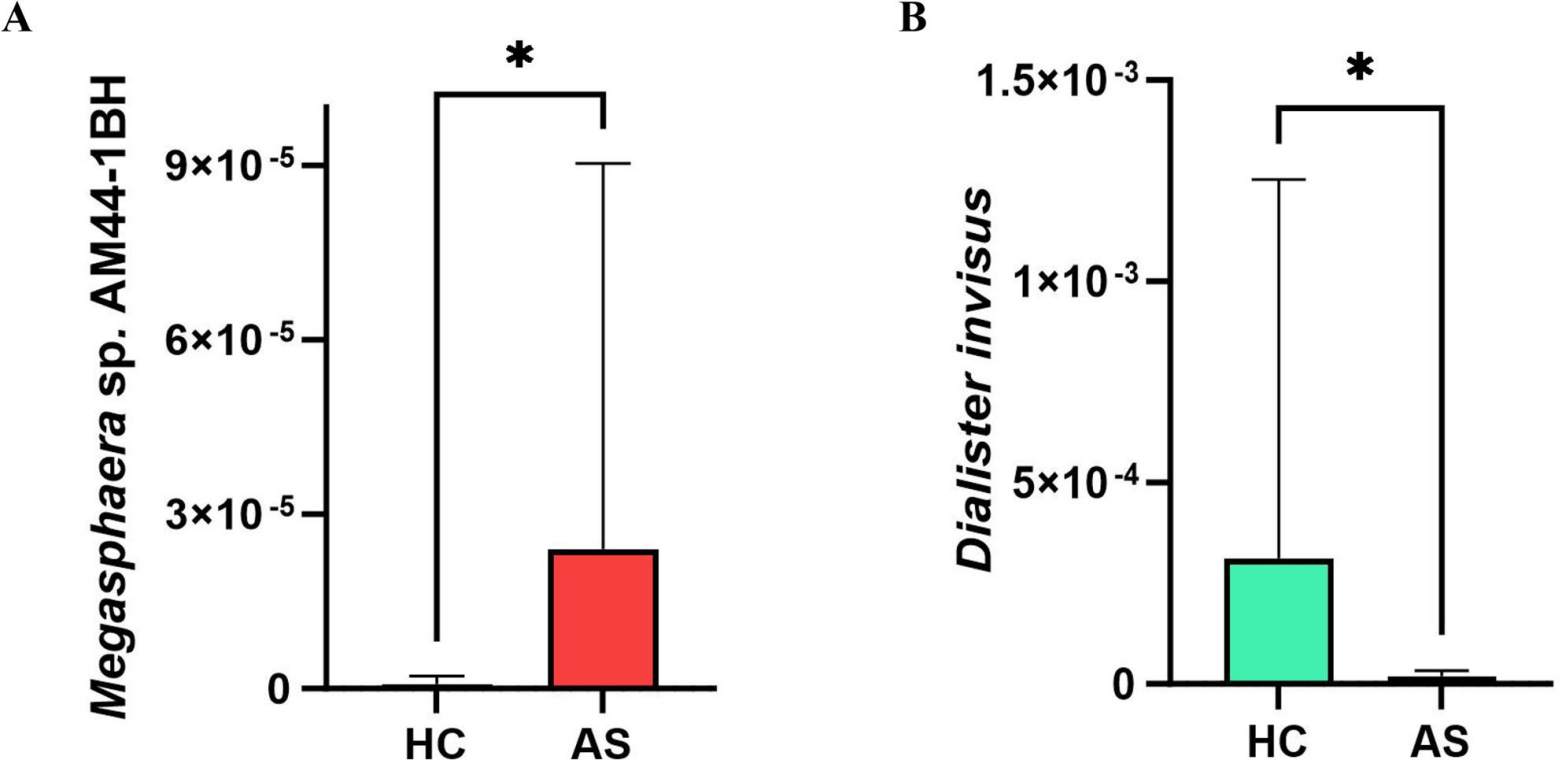
Relative abundances of key differential species between HC and AS groups. **A** *Megasphaera* sp.AM44-1BH is a species with significantly increased relative abundance at the species level in the AS group; **B** *Dialister invisus* is a species with significantly decreased relative abundance at the species level in the AS group

DESeq2 analysis was performed using the species-level absolute abundance tables from the HC and AS groups. A total of 43 significantly different species were identified (FDR < 0.05). Among these, 22 species showed higher abundance in the AS group compared to the HC group, suggesting potential pathogenic roles in AS, while 21 species showed lower abundance, suggesting potential probiotic roles in AS (Fig. 5). To further validate these differences, we conducted intergroup comparisons of their relative abundances. The results revealed that *Megasphaera* sp.AM44-1BH was significantly enriched in the AS group, whereas *Dialister invisus* was significantly depleted in the AS group; these two species were therefore selected as key differential species for presentation (Fig. 6A and B). Overall, other species with significantly increased abundance in the AS group included *Mitsuokella multacida* and *Sutterella* sp. KLE1602, while species with significantly decreased abundance in the AS group primarily comprised *Bifidobacterium bifidum* and *Blautia hansenii*, among others.

#### Species LEfSe analysis

**Fig. 7:**
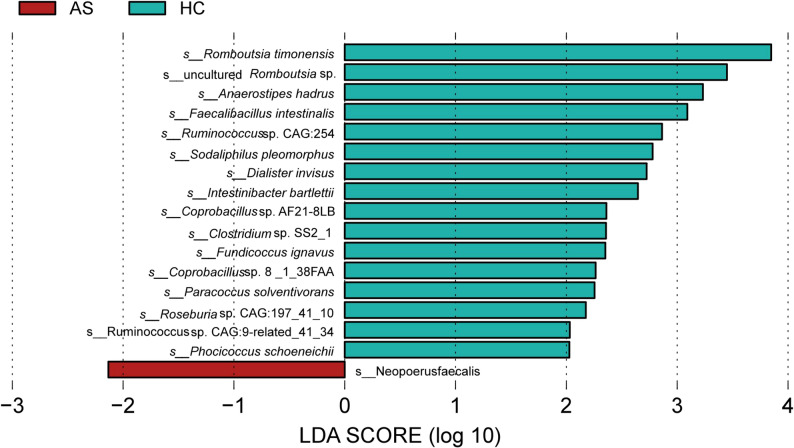
LDA value distribution map of intestinal microbiota at the species level

The HC group had 16 dominant species, among which *Romboutsia timonensis*, uncultured *Romboutsia* sp., *Anaerostipes hadrus*, and *Faecalibacillus intestinalis* had a greater impact on the abundance difference; the AS group had 1 dominant species (*Neopoerus faecalis*), among which *Neopoerus faecalis* had a greater impact on the abundance difference of grouping (Fig. 7).

### Functional annotation

#### Overview of functional relative abundance

**Fig. 8:**
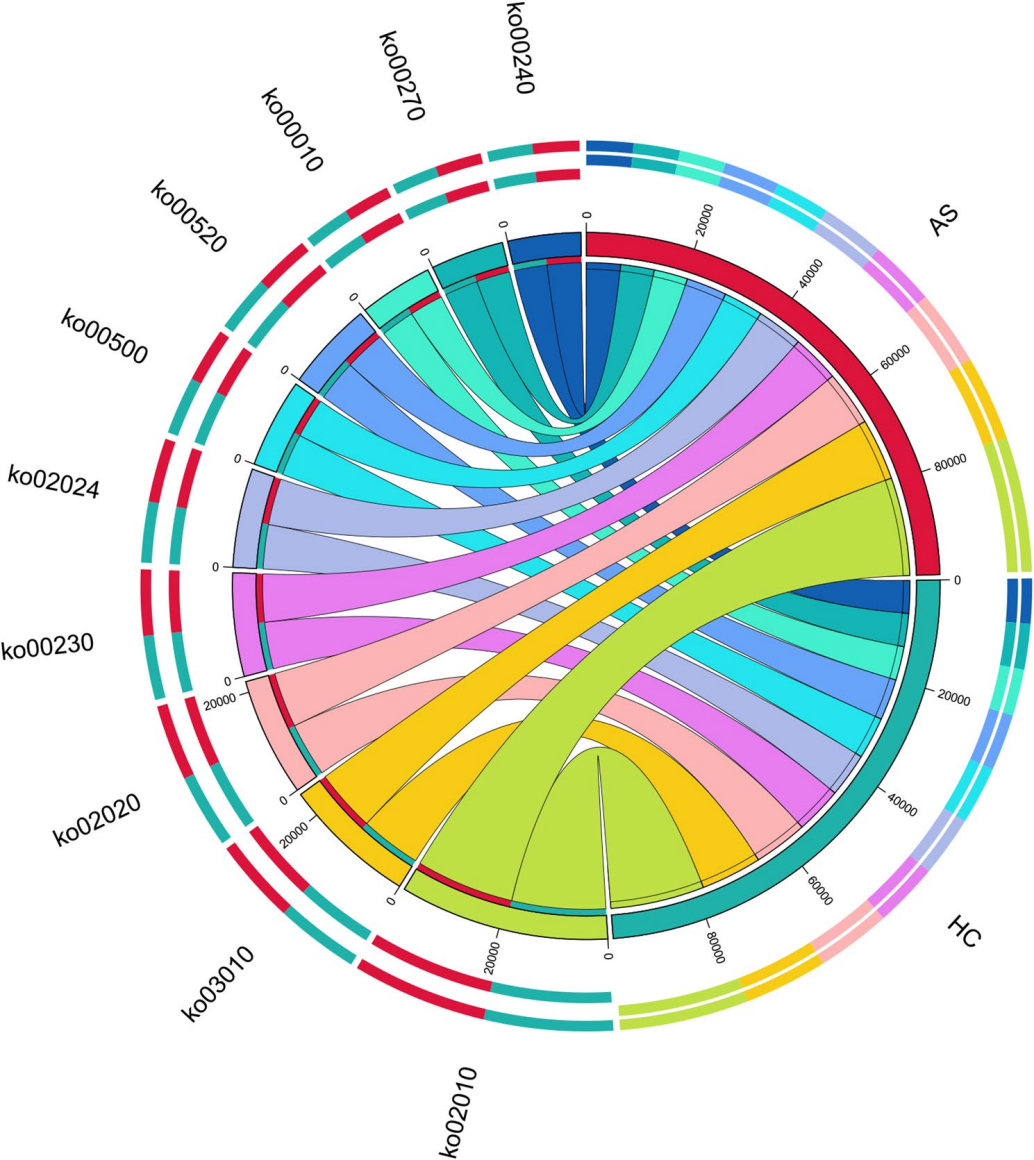
Relative abundance of the top 10 functions in the KEGG pathway database at level 3

At the KEGG PATHWAY level 1, the relative functional abundance of Metabolism was the highest he KEGG PATHWAY level 1, the relative functional abundance of Metabolism was the highest in both the AS and HC groups; at level 2, the relative functional abundance of Carbohydrate metabolism was the highest; at level 3, the top 10 functional genes in terms of relative abundance included ABC transporters (ko02010), Ribosome (ko03010), and Two-component system (ko02020). There were differences in the relative abundance of the top 10 functions in the KEGG PATHWAY database of intestinal microbiota between the two groups (only level 3 is shown in Fig. 8).

#### Functional PCoA analysis

**Fig. 9:**
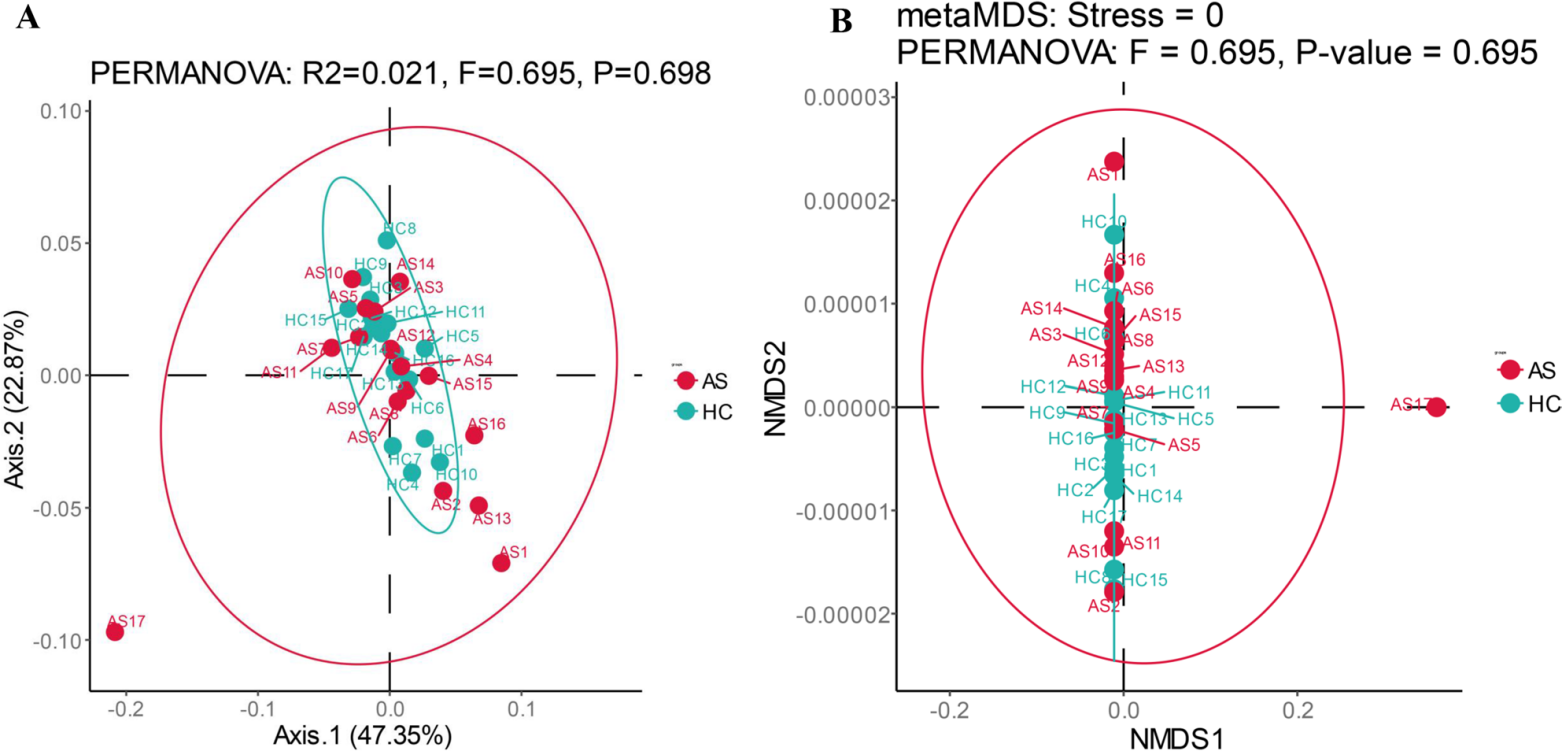
Functional analysis results of gut microbiota at level 3. **A** PCoA analysis; **B** NMDS analysis

PCoA analysis was conducted based on each functional level. The overlapping of the fitted circles between the AS group and the HC group was relatively high. At level 3, the overlapping rate of the fitted circles between the AS group and the HC group was relatively high, with R²=0.021 and *P* = 0.698, indicating no significant difference between the samples (*p* > 0.05) (Fig. 9A).

#### Functional NMDS analysis

NMDS analysis was conducted based on each classification level. Stress < 0.2 indicated that NMDS could accurately reflect the degree of difference between samples. At level 3, the overlapping rate of the fitted circles between the HC group and the AS group was relatively high, with p-value = 0.695, indicating no significant difference between the samples (*p* > 0.05) (Fig. 9B).

#### Functional LEfSe analysis

**Fig. 10:**
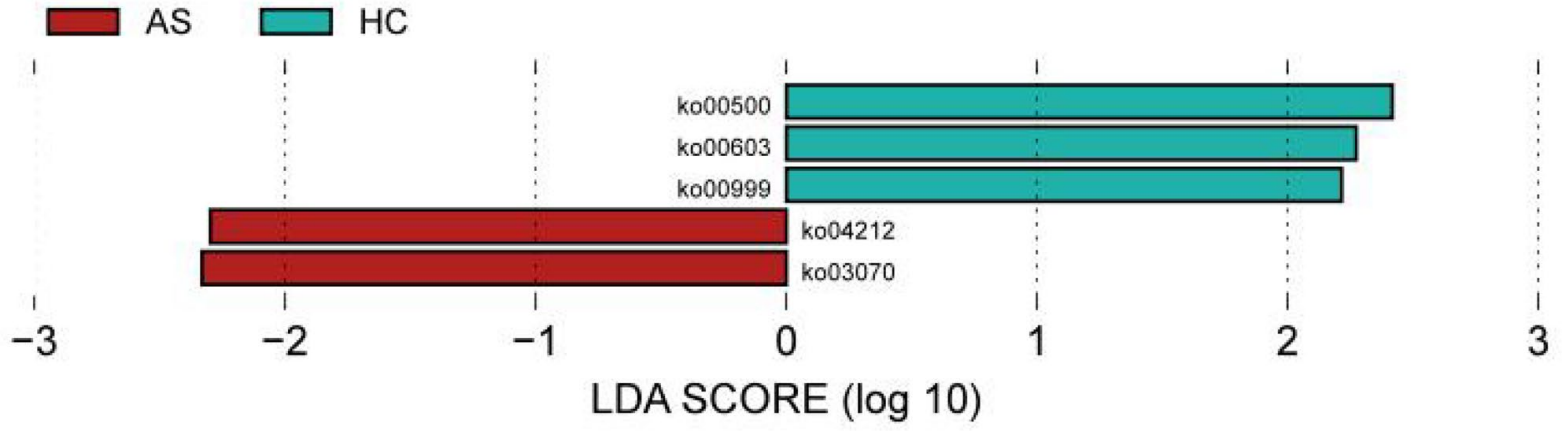
Functional LEfSe analysis results at level 3

When LDA > 2, at level 3, the dominant genes in the HC group included Starch and sucrose metabolism (ko00500), Glycosphingolipid biosynthesis (ko00603), and Biosynthesis of various plant secondary metabolites (ko00999); the dominant genes in the AS group included Longevity regulating pathway (ko04212) and Bacterial secretion system (ko03070) (Fig. 10). The differential metabolic pathways screened at level 3 were mainly concentrated in pathways such as Metabolism at level 1, and Carbohydrate metabolism, Lipid metabolism, and Biosynthesis of other secondary metabolites at level 2.

#### Functional characteristics of unique gene sets

**Fig. 11:**
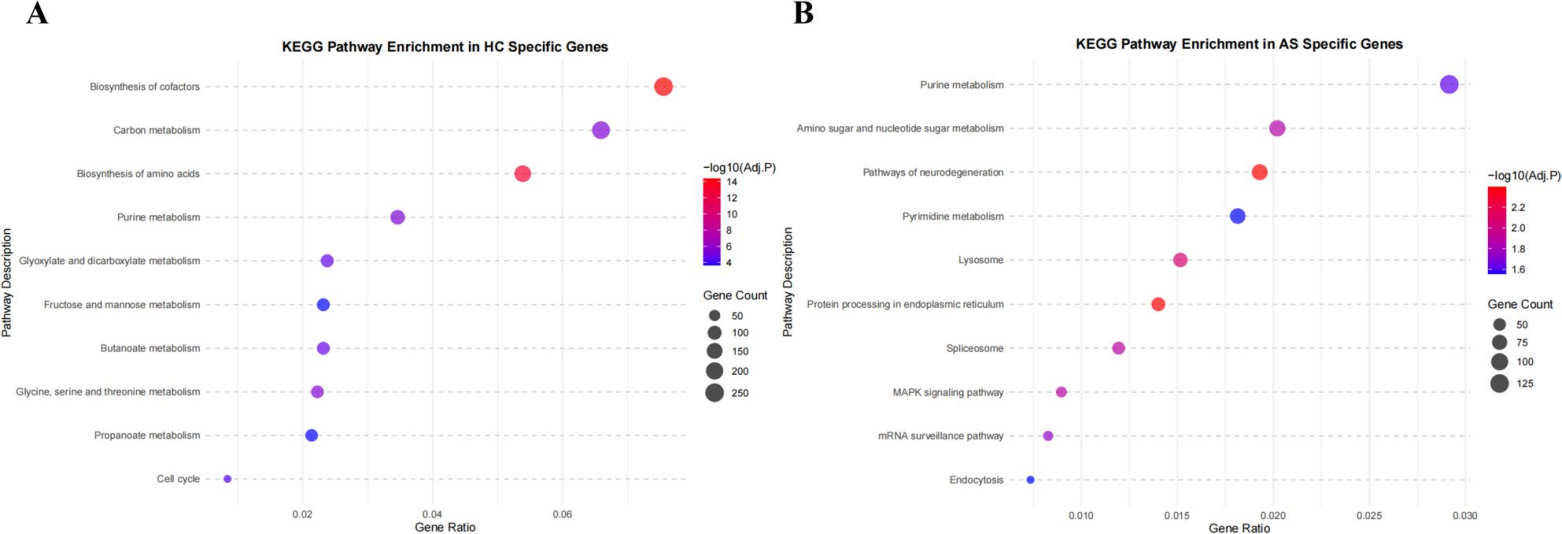
Top ten pathways from functional annotation of AS-group and HC-specific genes. **A** Top Ten Pathways of HC; **B** Top Ten Pathways of HC

KEGG pathway enrichment analysis was performed on the specific genes identified in the AS and HC groups. Among the 566,526 unique genes in the AS group, 147,372 (26.01%) were annotated with KEGG Orthology (KO) terms; among the 406,609 unique genes in the HC group, 152,781 (37.57%) received KO annotations. After excluding cancer-related and associated cellular pathways, the specific genes in the AS group were found to be primarily enriched in 13 pathways, including neurodegenerative disease pathways (ko05022), protein processing in the endoplasmic reticulum (ko04141), and purine metabolism (ko00230). The specific genes in the HC group were mainly enriched in 36 pathways, including cofactor biosynthesis (ko01240), amino acid biosynthesis (ko01230), and glycine, serine, and threonine metabolism (ko00260) (Fig. 11).

#### Microbial source tracking of AS-specific pathways

**Fig. 12:**
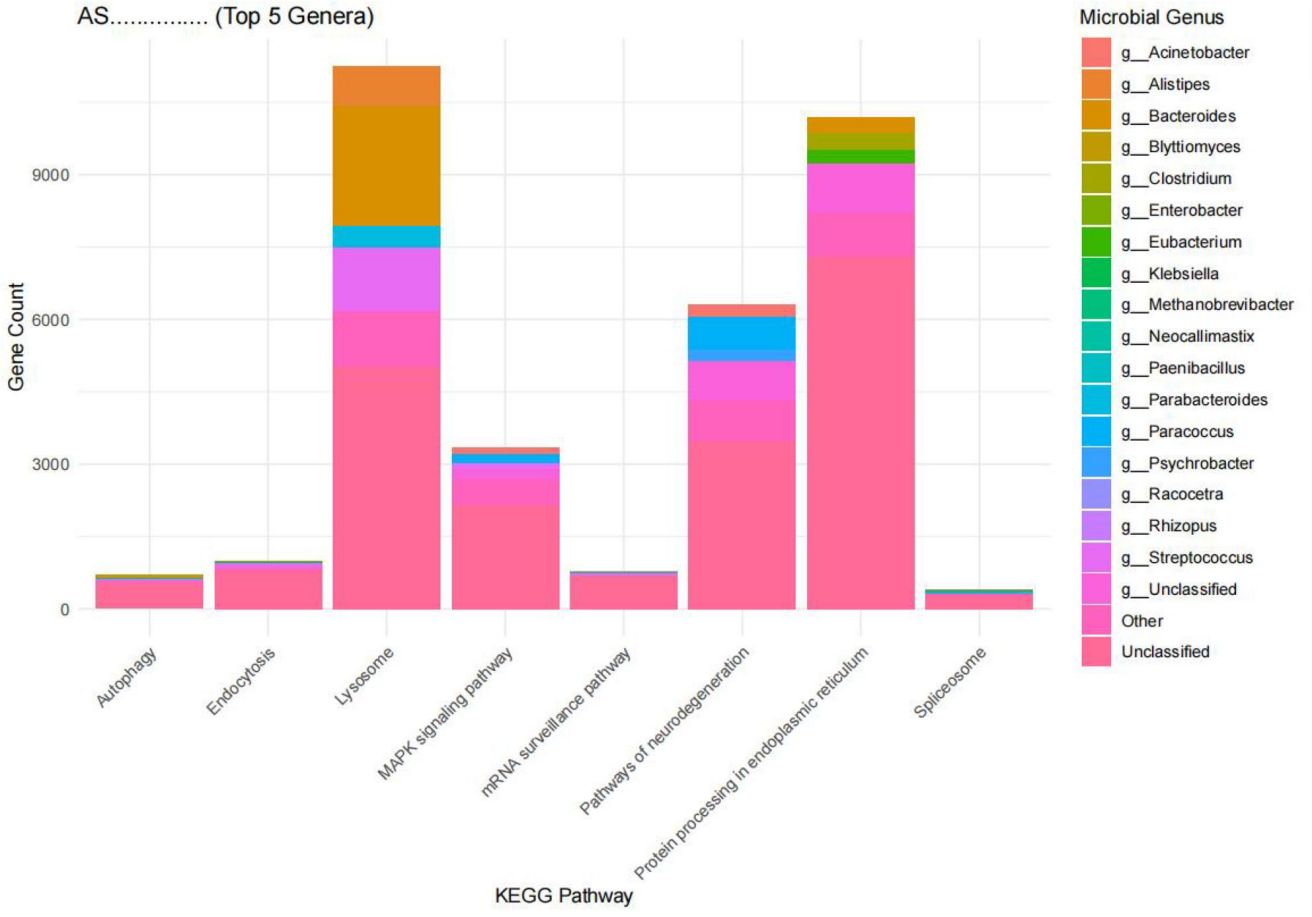
Microbial source tracking analysis results of AS-specific pathways

By comparing the 13 pathways identified in the AS group with those in the HC group, 8 AS-specific pathways were selected for microbial source tracking analysis. As shown in Fig. 12 unclassified genera contributed the highest proportion of genes, accounting for approximately 30%-80% of the total in each pathway. *Acinetobacter* was present in four pathways: mRNA surveillance, spliceosome, MAPK signaling, and neurodegeneration. *Bacteroides* appeared in the MAPK signaling, protein processing, lysosome, and neurodegeneration pathways. In the lysosome pathway, *Bacteroides* (18%) and *Streptococcus* (9.4%) occupied substantial proportions. The autophagy pathway was primarily influenced by *Eubacterium*, *Faecalibacterium*, *Roseburia*, and other genera (Fig. 12).

### Association analysis between microbial characteristics, function, and clinical scores

**Fig. 13:**
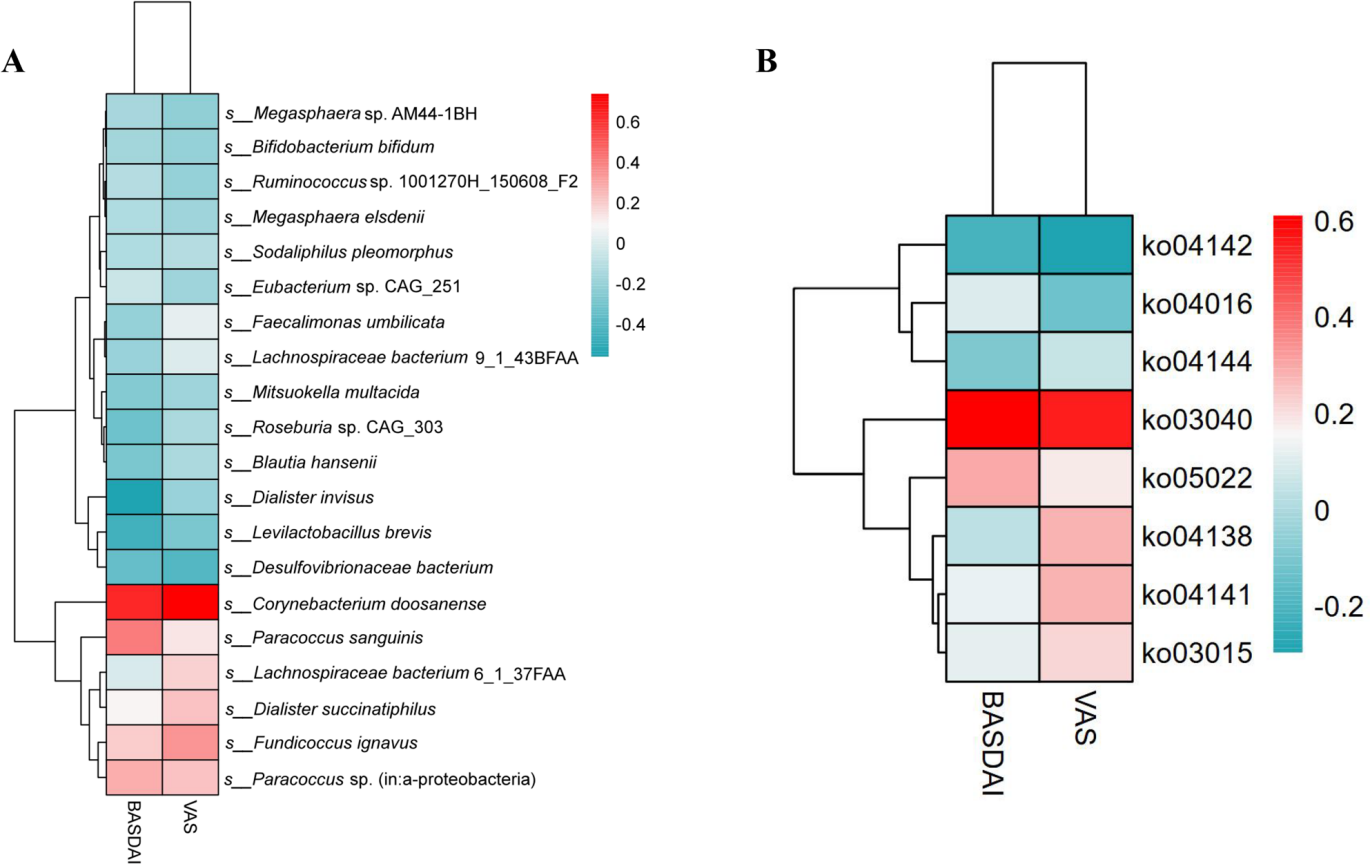
Association of differential microbial species and functional pathways with clinical scores in AS and HC groups. **A** Correlation analysis between intergroup differential species and clinical scores; **B** Correlation analysis between intergroup differential function and clinical scores

Through DESeq2 analysis, the relative abundance of differentially abundant species between groups was examined for Spearman rank correlation with BASDAI and VAS scores. After FDR correction, no species showed a significant association with any clinical score (all q > 0.05) (Fig. 13A).

To overcome the limitations of univariate analysis, we further employed a random forest model to assess the ability of the combination of differentially abundant species identified by DESeq2 to predict BASDAI scores grouped into high and low categories using a cutoff of 4. The model performed poorly, with a prediction accuracy of 35.3% and an area under the curve (AUC) of 0.625, indicating limited capacity to distinguish disease activity based on the current set of differential species.

KEGG was selected pathways specifically enriched in the AS group and evaluated their abundance for Spearman correlation with the aforementioned clinical scores. After multiple testing correction, no pathway was found to be significantly associated with clinical scores (all q > 0.05) (Fig. 13B).

## Discussion

This study analyzed the intestinal microbiome of AS patients and HC using metagenomic methods and found that although there was no significant difference in the overall microbial diversity between the two groups, there were obvious differences in the composition of specific bacterial species and functional potential, which was consistent with existing studies, suggesting that intestinal microorganisms may be involved in the pathogenesis of AS [14, 15].

At the genetic level, the number of unique genes in the AS group (566,526) was significantly higher than that in the HC group (406,609), reflecting that the intestinal microorganisms of AS patients may have a more specific gene set to adapt to the host’s inflammatory environment, and this genetic difference may be associated with changes in the metabolic function of microorganisms [16].

In terms of species diversity, there were no significant differences in the Alpha diversity indices (Chao1, Shannon, Simpson) at the species level or the overall microbial structure (PCoA, NMDS) (*p* > 0.05), which was inconsistent with some reports that the intestinal microbial diversity of AS patients is reduced [15]. This difference may be caused by differences in study populations, sample sizes, or methodologies; for example [14], found that the intestinal bacterial diversity in AS patients was reduced, but there was no significant change in diversity in this study, suggesting that the change in diversity may be less obvious in larger samples or different populations. At the phylum level, the dominant phyla were the same in both groups (*Bacillota*, *Bacteroidota*, etc.), and the abundance of *Pseudomonadota* in the AS group showed an increasing trend but was not significant; at the species level, the dominant species in both groups was *Segatella copri*, but its functional state may differ due to the host’s pathological state.

In terms of species abundance, there were significant differences at the family, genus, and species levels. Through DESeq2 analysis, we identified a total of 43 significantly differential species, with 22 species enriched (e.g., *Megasphaera* sp. AM44-1BH) and 21 species diminished (e.g., *Dialister invisus*) in the AS group. Notably, the most abundant species, *Segatella copri* (i.e., *Prevotella copri*), has frequently been reported to be enriched in AS and is often co-enriched with genera such as *Megasphaera*, suggesting they may play a synergistic role in the AS gut microenvironment [17].Furthermore, the diminished *Dialister* genus has been suggested by Mendelian randomization studies to have a protective effect against AS [18], while another enriched genus, *Sutterella*, has also been found to be enriched in patients with rheumatoid arthritis (RA), supporting the notion that certain microbes may share common roles across spondyloarthritis [19]. On the other hand, diminished species such as *Bifidobacterium bifidum* and *Blautia hansenii* are generally considered to possess probiotic or protective potential. The decrease in these anti-inflammatory-related species may be associated with intestinal barrier damage and aggravated inflammation [20]; LEfSe analysis showed that the HC group had 16 dominant bacteria, while the AS group had only *Neopoerus faecalis* as the dominant bacterium, which supports the “dysbiosis” hypothesis. Moreover, the HLA-B27 allele may promote the pathogenesis of AS by affecting the composition of intestinal microbiota [21, 22].

At the functional level, there were differences in KEGG level 3 functional pathways (such as ABC transporters and Ribosome pathways), involving nutrient transport, signal transduction, etc., which may be associated with microbial metabolism and host-microbe interactions. The HC group was enriched with functional genes related to carbohydrate metabolism; the AS group showed an increasing trend in the bacterial secretion system, which may suggest that the intestines of AS patients are under microecological stress [23–25]. However, functional PCoA and NMDS analyses showed no significant differences between the groups, which is due to the functional redundancy of microbial communities, where different bacterial species may perform similar functions to mask overall changes [24]. Beta diversity analyses (e.g., PCoA, NMDS) are sensitive to shifts in high-abundance taxa or broad functional features [26]. However, they may fail to detect subtle but biologically critical changes driven by low-abundance taxa or confined to specific functional modules. Due to functional redundancy, the overall community structure can remain stable despite these specific alterations, leading to a non-significant result in global analyses and potentially overlooking important biological signals [27].

The unique genes in the AS group were enriched in pathways related to neurodegenerative diseases, endoplasmic reticulum protein processing, and purine metabolism, whereas those in the HC group were enriched in basic metabolic pathways such as cofactor and amino acid biosynthesis [28]. This suggests that the gut microbiota of AS patients may be more involved in metabolic processes linked to inflammation and cellular stress. Microbial source tracking further linked some AS-enriched pathways (e.g., MAPK signaling, neurodegenerative diseases) to specific bacterial genera (e.g., Acinetobacter, Bacteroides) [29]. Notably, the enrichment of the “protein processing in endoplasmic reticulum” pathway is of particular interest, as literature indicates that misfolding of HLA-B27 molecules can lead to their accumulation in the endoplasmic reticulum, triggering endoplasmic reticulum stress [30]. Additionally, autophagy, another relevant pathway, is a crucial mechanism for clearing misfolded proteins, and its dysfunction has been implicated in autoimmune diseases including AS.

This study found no significant correlations between the abundance of these differential species or functional pathways and clinical scores (BASDAI, VAS) in AS, and the random forest model based on differential species showed limited predictive capacity for disease activity. This indicates that in the current study cohort, the identified microbial characteristics exhibited weak direct linear associations with clinical symptoms or disease activity [31]. This may be attributed to the fact that disease manifestations are regulated by multiple factors including genetics, immune status, and environment, with the microbiome representing only one component; alternatively, it may suggest that microbial influences primarily act at the levels of disease initiation or immune modulation, rather than directly determining the intensity of immediate clinical symptom [32].

A major limitation of this study is the relatively small sample size (*n* = 17 per group), which may lead to insufficient statistical power and affect the robustness and generalizability of the findings. This may also account for the negative results observed in the clinical correlation analyses. Future studies should seek to validate these findings in larger cohorts or multi-center designs to enhance the reliability of the conclusions.

## Conclusion

This study provides new potential evidence for the pathogenesis of ankylosing spondylitis (AS) from the perspective of gut microbiota. Compared with healthy individuals, the gut microbiota of AS patients showed no significant difference in overall diversity, but exhibited distinct differences in genetic specificity, abundance of specific bacterial species, and functional metabolic pathways. These differences suggest that intestinal microorganisms may be involved in the pathological process of AS, particularly as the pathways enriched by their unique genes show potential links to known immune mechanisms of AS. However, due to the limited sample size of this study, no direct correlation was observed between the identified microbial characteristics and clinical disease activity. Future research should further expand the sample size and integrate multi-omics data such as metagenomics and metabolomics, along with longitudinal study designs, to validate the current findings, elucidate the specific mechanisms of gut microbiota in AS, and thereby lay the foundation for developing microbiota-targeted diagnostic or interventional strategies.

## Supplementary Information

Supplementary Material 1.

## Data Availability

The original contributions presented in the study are included in the article/Supplementary Material. The raw sequence data reported in this paper have been deposited in the Genome Sequence Archive [33] in National Genomics Data Center [34] , China National Center for Bioinformation / Beijing Institute of Genomics, Chinese Academy of Sciences ( **GSA: CRA032090** ) that are publicly accessible at https://ngdc.cncb.ac.cn/gsa.further inquiries can be directed to the corresponding author.
