# Supplementary material for: Metagenomic analysis of intestinal microbiota characteristic differences between patients with ankylosing spondylitis and healthy individuals
Source: BMC Microbiol. 2026 Apr 6;26:472. doi: 10.1186/s12866-026-04996-8 (PMC13191970; doi:10.1186/s12866-026-04996-8)
Supplement: Supplementary file 1 — Supplementary Material 1. [file 12866_2026_4996_MOESM1_ESM.docx]

**Supplementary Material**

**Metagenomic Analysis of Intestinal Microbiota Characteristic Differences Between Patients with Ankylosing Spondylitis and Healthy Individuals**

Shuo-wen Liu ^1, a^, Xin-xin Wang ^1, a^, Le-yao Xian ^2^, Da-wei Zou ^2^, Yu-feng Huang ^2^, Xi-lin He ^3^, Fan He ^2, *^, Xiao-tong Wang ^1, *^

1. The First Clinical College of Liaoning University of Traditional Chinese Medicine, Affiliated Hospital of Liaoning University of Traditional Chinese Medicine, Shenyang, Liaoning, China.
2. Guangdong Provincial Hospital of Chinese Medicine, The Second Affiliated Hospital of Guangzhou University of Chinese Medicine, Guangzhou, Guangdong, China.
3. Faculty of Arts & Science, University of Toronto, St. George Campus, Toronto, Ontario, Canada.

^a^ These authors have contributed equally to this work.

* Correspondence authors

Fan He: hefan_78@126.com, Xiao-tong Wang: wangxt1116@163.com

**Supplementary**

**Recruitment of Study Subjects**

1. Inclusion Criteria for Patients

- Aged over 18 years, regardless of gender;
- Meet the 1984 revised New York Diagnostic Criteria for Ankylosing Spondylitis (AS) ^[1]^ or the Classification Criteria for Radiographic Axial Spondyloarthritis (axSpA) formulated by the Assessment of SpondyloArthritis International Society (ASAS) in 2009, with imaging examination results;
- Patients in the active stage who meet the Western medicine diagnostic criteria for AS [Bath Ankylosing Spondylitis Disease Activity Index (BASDAI ≥ 4 ^[2]^, Visual Analogue Scale (VAS≥ 40/100] ^[3]^.

1. Exclusion Criteria for Patients

- Patients diagnosed with other rheumatic immune diseases;
- Patients with other combined rheumatic diseases;
- Patients who have used rheumatic immune inhibitors or non-steroidal anti-inflammatory drugs within the past 3 months;
- Patients with completely rigid or curved/deformed spine in the advanced stage, severe deformity or disability, and sacroiliitis of radiological grade IV;
- Participants who have received treatment with antibiotics, traditional Chinese medicine, or any products containing active bacterial components within the past 4 weeks, as well as those with constipation or diarrhea before sampling;
- Abnormal laboratory indicators in baseline examination: platelet count < 100×10⁹/L; white blood cell count < 3.7×10³/mm³; polymorphonuclear leukocyte count < 2×10³/mm³; hemoglobin < 100mg/L; serum creatinine > 133μmol/L; AST, ALT, ALP, GGT, and TBIL higher than 1.25 times the normal value; abnormal urine routine, red blood cells > 0/HP in stool routine, and positive occult blood;
- Patients with combined severe heart, liver, brain, lung, kidney, or hematological system diseases.

1. Inclusion Criteria for Healthy Controls

- Aged ≥ 18 years, regardless of gender;
- Able to cooperate with blood and fecal sample collection;
- Voluntarily sign the informed consent form.

1. Exclusion Criteria for Healthy Controls

- No severe physical diseases;
- No history of gastrointestinal diseases (such as inflammatory bowel disease), rheumatic diseases, or other chronic internal diseases.

**References**

1. Van der Linden S, Valkenburg HA, Cats A. Evaluation of diagnostic criteria for ankylosing spondylitis. A proposal for modification of the New York criteria. Arthritis and rheumatism. 1984; 27(4): 361–368. doi:10.1002/art.1780270401
2. Zochling J. Measures of symptoms and disease status in ankylosing spondylitis: Ankylosing Spondylitis Disease Activity Score (ASDAS), Ankylosing Spondylitis Quality of Life Scale (ASQoL), Bath Ankylosing Spondylitis Disease Activity Index (BASDAI), Bath Ankylosing Spondylitis Functional Index (BASFI), Bath Ankylosing Spondylitis Global Score (BAS-G), Bath Ankylosing Spondylitis Metrology Index (BASMI), Dougados Functional Index (DFI), and Health Assessment Questionnaire for the Spondylarthropathies (HAQ-S). Arthritis care & research. 2011;63 Suppl 11:S47–S58. doi:10.1002/acr.20575
3. Johnson EW. Visual analog scale (VAS). American journal of physical medicine & rehabilitation. 2001;80(10):717. doi:10.1097/00002060-200110000-00001
